# Optical design of nanowire absorbers for wavelength selective photodetectors

**DOI:** 10.1038/srep15339

**Published:** 2015-10-15

**Authors:** S. Mokkapati, D. Saxena, H. H. Tan, C. Jagadish

**Affiliations:** 1 Department of Electronic Materials Engineering, Research School of Physics and Engineering, The Australian National University, Canberra, A. C. T. 2601, Australia

## Abstract

We propose the optical design for the absorptive element of photodetectors to achieve wavelength selective photo response based on resonant guided modes supported in semiconductor nanowires. We show that the waveguiding properties of nanowires result in very high absorption efficiency that can be exploited to reduce the volume of active semiconductor compared to planar photodetectors, without compromising the photocurrent. We present a design based on a group of nanowires with varying diameter for multi-color photodetectors with small footprint. We discuss the effect of a dielectric shell around the nanowires on the absorption efficiency and present a simple approach to optimize the nanowire diameter-dielectric shell thickness for maximizing the absorption efficiency.

III-V semiconductor nanowires exhibit strong waveguiding properties for wavelengths below the bandgap wavelength despite their small cross section (few 100 s of nm) because of high refractive indices^1^. The waveguiding properties of nanowires result in absorption and emission characteristics very different to the same material in bulk, planar configuration^2^,^3^,^4^. Several interesting phenomenon have been observed in optoelectronic devices fabricated from nanowires due to their waveguiding properties. For e.g., nanowire lasers exhibit power confinement factors^1^ larger than 1, nanowire solar cells have the potential to achieve higher V_oc_ and efficiency compared to planar solar cells^5^ and nanowire photodetectors exhibit resonant peaks in responsivity^6^.

For optoelectronic devices like solar cells and photodetectors that convert light into electrical signal, the waveguide modes supported in the nanowire offer the possibility of tailoring the absorption properties. This is an additional advantage over other nanoscale photodetectors fabricated from 2D materials that have received a lot of attention in recent times^7^,^8^. Effect of guided modes on nanowire photodetector performance characteristics has already been discussed for devices illuminated with light propagating in a direction perpendicular to the axis of the nanowire (horizontal configuration)^6^. In vertical illumination configuration i.e., incident light propagating along the direction parallel to the axis of the nanowire (Fig. 1(a)) the resonant modes to which incident light couples are different to the resonant modes that alter absorption characteristics of nanowires in the former configuration. Also different projected areas lead to very different absorption characteristics in the nanowires. For e.g., in horizontal configuration, absorption efficiency^5^ is ~1 but in vertical configuration, absorption efficiency can be very large^9^. In this paper, we discuss the implications of guided modes on the performance characteristics of nanowire photodetectors in the vertical configuration.

For fabrication of photodetectors a dielectric shell is often required to isolate the two device contacts. There have been reports in the literature claiming that a dielectric shell can lead to increased absorption in the nanowire^10^,^11^. All these reports have compared integrated absorption over the entire solar spectrum in a horizontal nanowire with and without a dielectric shell for solar cell applications. Dielectric shell around the nanowire shifts the spectral position of peak absorption. For solar cell applications the shift in spectral position of peak absorption is not critical as long as the integrated absorption is maximized. However, shift in peak absorption position needs to be considered for wavelength selective photodetectors. Indeed, we present an approach to determine the optimal nanowire diameter and shell thickness for maximizing light absorption in vertical nanowires and discuss the implications of the presence of a dielectric shell on photodetector performance.

In this study nanowires with circular cross section are illuminated with a plane wave propagating in a direction parallel to the axis of the nanowire, with electric field vector oscillations (polarization) in the radial direction. InP nanowires are very promising for optoelectronic devices since they have a relatively small surface recombination velocity^12^ compared to other III-V compounds. For this reason, we chose InP nanowires as an example. The length of the nanowires is fixed at 4 μm for this study considering feasibility of fabrication for future experimental studies. The length of the nanowire, however does not affect the resonant modes of interest to this manuscript. All the concepts presented in the rest of the manuscript using these nanowires, are valid irrespective of the actual length.

Figure 1(b) shows the absorption cross section of a single InP nanowire as a function of nanowire diameter and incident wavelength. The data is obtained by numerically solving the Maxwell’s Equations using Lumerical FDTD Solutions package^13^. The nanowire diameter is varied between 100 and 400 nm. For these diameters the maximum absorption cross section for the nanowires is ∼2 μm^2^ for wavelengths just below the bandgap wavelength of InP. The absorption cross section data shows distinct branches which are a consequence of the waveguiding properties of nanowires.

As discussed earlier, III-V semiconductor nanowires behave as waveguides due to their high refractive index and aspect ratio. They support waveguide modes propagating along the nanowire axis. Light incident on the nanowires can be coupled to the resonant modes supported in the nanowires. The efficiency of coupling incident light to the resonant waveguide modes supported in the nanowire depends on the overlap of the incident fields and the field profile of the guided modes. For the illumination configuration shown in Fig. 1(a), the overlap is non-zero only for HE_1n_ modes^4^,^14^,^15^,^16^,^17^. Once incident light is coupled to these modes, the efficiency with which it is absorbed in the nanowire is determined by how strongly the fields are confined to the nanowire. Thus, to maximize light absorption in the nanowire, the product of the overlap integral between incident field and the guided mode, and confinement factor for the guided mode should be maximized. The dashed lines in Fig. 1(b) show the maxima of the product of the overlap integral between the incident field and the guided mode, and the confinement factor for HE_11_, HE_12_ and HE_13_ modes. Figure 1(c) shows the electric field intensity profiles across the cross section of a nanowire with a diameter of 125 and 320 nm for an incident wavelength of 700 nm. The field intensity profile matches very well with HE_11_ and HE_12_ modes, respectively. The field intensity profiles across the nanowire cross section and the very good match between the position of the dashed lines with the strong absorption branches in the absorption cross section data, confirm that the absorption properties of the nanowires are indeed due to coupling of incident light into HE_1n_ guided modes supported in the nanowires.

The absorption efficiency data for the nanowires is shown in Fig. 2(a). The data is obtained by dividing the absorption cross section by the nanowire projected area 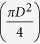, where *D* is the diameter of the nanowire. Small diameter nanowires have very high absorption efficiency and for larger diameters the absorption efficiency reduces due to the increase in nanowire projected area. For 4 μm long InP nanowires, maximum absorption efficiency is ∼140 at an incident wavelength of 600 nm for nanowire diameter of 100 nm. As discussed earlier, the high absorption efficiency for small diameter nanowires is due to coupling of incident light into HE_11_ guided mode supported in the nanowires. The absorption branch due to in-coupling of light into HE_12_ guided mode is also visible for large diameter nanowires, but has a much lower magnitude.

Large absorption efficiency and shift in spectral position of the absorption efficiency with nanowire diameter can be exploited to realize wavelength selective photodetectors with small foot-print using the same semiconductor material. The shift in the spectral position of absorption efficiency with nanowire diameter will lead to spectral shift in the peak response of the nanowire photodetectors with diameter. The absorption efficiency peak position in small diameter nanowires (100–150 nm) can be tuned between 600 nm and just below the bandgap of InP by controlling the nanowire diameter as shown in Fig. 2(b). As discussed earlier small diameter nanowires have very high absorption efficiency due to coupling of incident light into the HE_11_ guided mode. For nanowire diameters larger than 200 nm, absorption is predominantly due to coupling of incident light into the HE_12_ resonant mode and the absorption efficiency is lower due to increase in the nanowire diameter. Absorption peak position in the nanowires varies with nanowire diameter even though the magnitude of absorption efficiency is reduced, as shown in Fig. 2(c).

The magnitude of the absorption efficiency in the nanowire represents the ‘area’ over which the nanowire absorbs incident light. The nanowire absorbs light that is incident on an area, 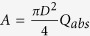, where *Q*_*abs*_ is the absorption efficiency. Greater than 1 absorption efficiency (*Q*_*abs*_) indicates that the nanowire absorbs light incident on an area larger than its projected area. The absorption efficiency for a bulk, planar semiconductor is ≤1. Hence a planar semiconductor with projected area *Q*_*abs*_ times larger than the projected area of a nanowire would be required to absorb the same power. The active semiconductor volume in a nanowire photodetector to achieve same photocurrent as in a planar photodetector is thus reduced by a factor *Q*_*abs*_, assuming that all the photogenerated carriers generate current in the external circuit. It should be noted here that a nanowire would not require any anti-reflection layers like in the case of a planar photodetector to maximize absorption. The absorption in nanowires is enhanced due to strong field confinement inside the nanowire at the spectral position where a resonant mode is supported.

Since the spectral position of maximum absorption efficiency in the nanowire changes with diameter, nanowires of varying diameter can be grouped together to realize multi-color photodetectors. Figure 3(a) shows the variation in peak absorption cross section for nanowires with a diameter of 100 and 150 nm as a function of the separation between the nanowires, s. The horizontal lines show the peak absorption cross section for the individual nanowires (i.e., s → ∞). For very small separation between the nanowires, they behave as a coupled system and the absorption cross section is lower than that for individual nanowires. The absorption peaks are also shifted in wavelength with respect to the peak position in individual nanowires as shown in Fig. 3(b). Each nanowire affects the absorption properties in adjacent nanowire. As the separation between the nanowires increases the absorption cross sections for the grouped nanowires converge to the absorption cross section for individual nanowires. Indeed, the absorption peak also converges to the peak position in individual nanowires and corresponds to a wavelength of 585 and 825 nm, for the smaller and larger diameter nanowires respectively (Fig. 3(b)). These results indicate that multi-color focal plane array photodetectors can be realized by grouping several nanowires with different diameters separated from each other by few hundreds of nm in each pixel element. The nanowires can be grouped together in any pattern, as long as the separation between adjacent nanowires is larger than the minimum requirement discussed above. The small separation required between individual nanowires makes this design ideal for focal plane array photodetectors which require very small footprint.

For device applications, a dielectric shell is often used around the nanowire to isolate the electrical contacts. The waveguiding properties of nanowires arise due to the high index contrast between the nanowire and the surrounding medium. Changing the index contrast between the nanowire and its surrounding medium by inserting a dielectric shell will change its waveguiding properties. As we have discussed earlier the absorption properties of the nanowires are a result of efficient coupling of incident light to the guided modes and confinement of the guided modes within the nanowire. The dielectric shell around the nanowire will affect both of these properties and hence change the absorption characteristics of the nanowire.

First we discuss the effect of a dielectric shell on the waveguiding properties of the nanowire. The effective index, n_eff_ of a mode supported in the nanowire describes how well the mode is confined in the nanowire. The real part of n_eff_ varies between the refractive index of the nanowire for very well confined modes and the index of the surrounding medium at cut-off. Figure 4(a) shows real(n_eff_) of the fundamental (HE_11_) mode as a function of nanowire diameter with and without a dielectric shell at the wavelength of 700 nm. Data is shown for a dielectric shell with index 1.8 and thickness 40 nm and a shell with index 2 and thickness of 20 or 40 nm. The imaginary component for the index of the dielectric shell is assumed to be 0. SiO_2_, Si(O)N, TiO_2_ are examples of dielectrics without absorption in the wavelength range of interest for InP nanowires. The real part of refractive index for these dielectrics varies between 1.3–2 and depends on the deposition technique. For a bare nanowire with diameter < 120 nm, real(n_eff_) is ∼1 indicating that the mode has large evanescent fields. Large evanescent fields enhance in-coupling of incident light to the guided modes supported in the nanowire due to better overlap of the field components. A dielectric shell around the nanowire increases real(n_eff_) since the ‘effective’ diameter of the nanowire is now larger. Figure 4(b) shows the mode profiles of the HE_11_ mode in a bare nanowire of diameter 120 nm without (top panel) and with (bottom panel) a dielectric shell, together with the complex n_eff_. Figure 4(a,b) show that with a dielectric shell, real(n_eff_) of the mode increases and hence smaller diameter nanowire is required for better in-coupling of incident light. The imaginary part of n_eff_ is also larger for the nanowire with the dielectric shell, and describes the propagation loss for the mode. Higher propagation loss for the resonant mode in the nanowire with the dielectric shell may result from stronger absorption in the nanowire due to better confinement.

There have been reports in the literature of dielectric shells improving the absorption characteristics of nanowires. All these reports have looked at the absorption characteristics in a nanowire in horizontal illumination configuration. To gauge the effect of dielectric shells on absorption characteristics of a vertical nanowire and its effect on the performance of wavelength selective nanowire-based devices it is necessary to compare the global maximum in the absorption of a bare nanowire to the global maximum in the absorption of a nanowire with a shell, at a given wavelength. However, this requires scanning a huge parameter space of nanowire diameter and dielectric shell thickness, which makes the task impractical. We now discuss a relatively simple approach to identify the optimal nanowire-shell parameters for maximizing absorption in a nanowire, compare the absorption characteristics of a bare nanowire with that of a nanowire with a dielectric shell and discuss the implications for photodetector applications.

As discussed earlier the maximum in absorption in a nanowire corresponds to the maximum of the product of overlap integral and confinement factor. We can evaluate this product for the guided modes in a bare nanowire and a nanowire with a dielectric shell to identify the optimal nanowire dimensions for maximum absorption. This is computationally less intensive and can be done either analytically or numerically using 2D simulations. This avoids the need to scan the entire parameter space of nanowire diameter and shell index/thickness to maximize nanowire absorption using computationally intensive 3D FDTD simulations. Figure 4(c) shows this data for a shell of index 1.8. As is expected from the guided mode behavior shown in Fig. 4(a), increasing shell thickness requires smaller nanowire diameter for maximizing absorption due to the increase in real(n_eff_) for a given nanowire diameter. As the dielectric shell thickness increases the overlap between the guided mode and the incident field increases, but the mode confinement inside the nanowire reduces as a result of lower index contrast between the nanowire and the surrounding medium. Thus there exists an optimal shell thickness for maximizing absorption that can be identified using this method.

Based on the above arguments direct comparison of absorption in a nanowire of fixed diameter with and without shell, which has been done in all reports to date, is not appropriate since the optimal nanowire dimensions are different for maximizing absorption without or with a dielectric shell. Table 1 shows the maximum absorption cross section and the corresponding absorption efficiency at 700 nm for a bare nanowire and a nanowire with a dielectric shell of index 1.8 or 2. The optimal nanowire diameter and the shell thickness for maximizing absorption are also listed. The absorption efficiency is determined by normalizing the absorption cross section to the nanowire core projected area. The area of dielectric shell is not included as it does not contribute to the generation of photocarriers. The absorption efficiency is maximum for a nanowire with a diameter of 115 nm and 15 nm dielectric shell of index 2, and exceeds that of a bare nanowire (125 nm) by 18.9%. The maximum absorption efficiency for a nanowire with a shell of index of 1.8 is reduced compared to this value, but is still higher than the maximum absorption efficiency in the bare nanowire by 10.5%.

To highlight the importance of optimizing the nanowire-shell dimensions for device applications we also evaluate the absorption efficiency for non-optimal nanowire-shell dimensions. Our calculations show that the absorption efficiency for a nanowire of diameter 120 nm and a shell thickness of 20 nm (index = 1.8) is reduced to 95.6. The absorption efficiency further reduces to 89 by increasing the shell thickness to 60 nm. Figure 4(c) shows that there is only a narrow range of optimal dimensions to maximize the absorption efficiency. This has implications for nanowire photodetectors and shows that the entire device structure needs to be properly designed for optimal optical performance. With proper optimization, a dielectric shell can enhance the photocurrent generated in a nanowire photodetector but non-optimal shell thickness may considerably degrade the nanowire device performance.

## Conclusions

The diameter-dependent resonant waveguide modes supported in III-V semiconductor nanowires enable design of photodetectors with wavelength tunable peak response, without having the need to change the material of the nanowire. Nanowire photodetectors have the potential to reduce the active semiconductor volume by a factor equal to the nanowire absorption efficiency compared to planar photodetectors fabricated from the same semiconductor material. Nanowires with varying diameters and wavelength scale separation between them can be used to realize multi-color focal plane array photodetectors with very small footprint. For practical devices it is necessary to identify the optimal nanowire diameter and thickness of the dielectric shell used for isolating the electrical contacts to maintain these advantages. We have illustrated a simple approach to optimize these parameters without having the need to scan a huge parameter space with computationally intensive approaches.

## Methods

### Simulations

The data presented in the manuscript was calculated using Lumerical^13^ FDTD solutions and MODE solutions packages. Three dimensional FDTD simulations were used for absorption calculations. The nanowire was illuminated with a broadband plane wave source and a three dimensional box of power monitors around the nanowire was used to determine the power inflow into the box. Wavelength dependent complex refractive index was used for the nanowire. Perfectly matched layer (PML) boundaries are used to minimize reflections from boundaries for calculating absorption in the nanowires. Two dimensional FDTD simulations were used for obtaining the mode profiles. The required mode was launched inside an infinitely long nanowire and the electric field intensity was recorded by placing a field monitor across the nanowire cross-section.

The dispersion relationship, confinement factor and the overlap integral were calculated using MODE solutions package. The confinement factor, Γ, for a mode propagating along the axis of the nanowire 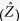 was calculated using 
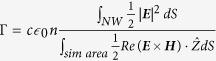
, where ***E*** and ***H*** are the electric and magnetic field vectors of the mode, *c* is the velocity of light in vacuum, *ε*_0_ is the permittivity of free space and *n* is the refractive index of the nanowire. The overlap integral between the resonant mode supported in the nanowire and the incident plane wave was calculated using 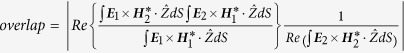, where the subscript 1 denotes the resonant mode and the subscript 2 denotes the incident plane wave.

## Additional Information

**How to cite this article**: Mokkapati, S. *et al.* Optical design of nanowire absorbers for wavelength selective photodetectors. *Sci. Rep.* **5**, 15339; doi: 10.1038/srep15339 (2015).

## Figures and Tables

**Figure 1:**
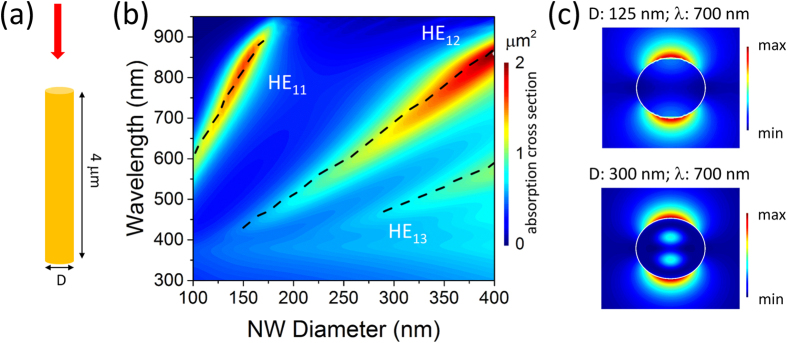
(**a**) Schematic illustration of the illumination configuration studied: A single nanowire is illuminated with a plane wave propagating along a direction parallel to the axis of the nanowire. The nanowire height is 4 μm. (**b**) Absorption cross section data for the nanowire as a function of its diameter and incident wavelength. The dashed lines are the maxima of product of overlap integral between incident field and field profile of the HE_11_, HE_12_ and HE_13_ modes and the corresponding confinement factors. (**c**) Electric field intensity profiles across the cross section of the nanowire at a wavelength of 700 nm for nanowire diameters of 125 nm and 300 nm.

**Figure 2:**
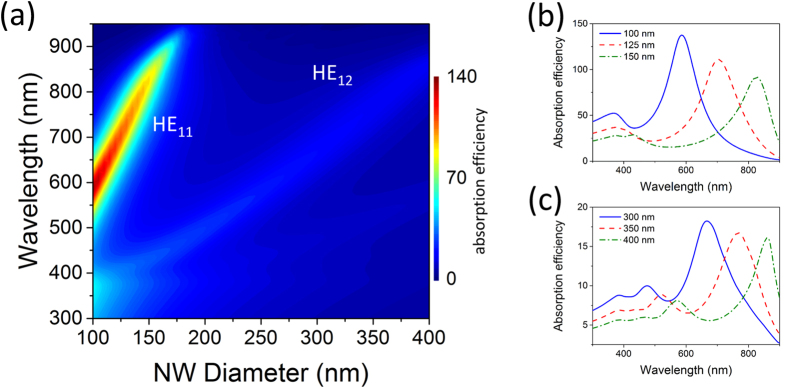
(**a**) Absorption efficiency as a function of incident wavelength for nanowire diameter varying between 100 nm and 400 nm. (**b**,**c**) Absorption efficiency vs. incident wavelength for three different nanowire diameters: absorption efficiency data shown in (**b**,**c**) is due to coupling of incident light to HE_11_ and HE_12_ guided modes, respectively.

**Figure 3:**
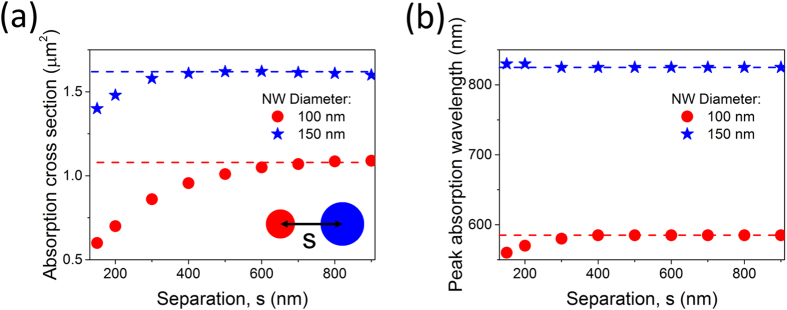
Variation in (a) absorption cross section and (b) peak absorption wavelength for 100 nm and 150 nm diameter nanowires, as a function of the separation between the nanowires. The inset in (a) shows the top view of the nanowires and defines the separation, s. The horizontal lines represent the absorption cross section or peak absorption wavelength for the individual nanowires (s → ∞).

**Figure 4:**
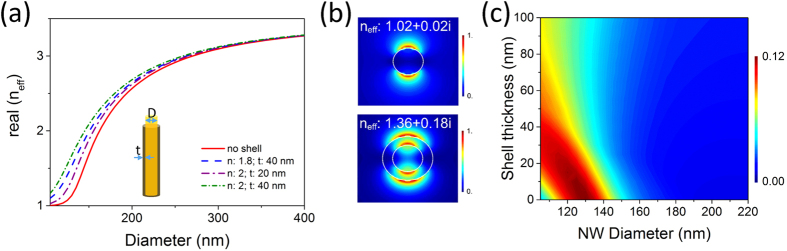
(**a**) Dispersion relation for HE_11_ mode at 700 nm for a bare nanowire and a nanowire with a dielectric shell. The x-axis is the diameter of the nanowire, shell thickness is not included. (**b**) HE_11_ mode profile in a nanowire of diameter 120 nm without (top) and with a dielectric shell of index 1.8 and thickness 40 nm (bottom). The white circles indicate the nanowire and shell positions. The complex effective indices are also indicated. (**c**) Product of confinement factor and overlap integral as a function of nanowire diameter and dielectric shell thickness (refractive index of the shell: 1.8).

**Table 1:** Maximum absorption cross section and the corresponding absorption efficiency at 700 nm for a bare nanowire and a nanowire with a dielectric shell.

NW Diameter (nm)	Shell index/thickness (nm)	Absorption cross section (μm^2^)	Absorption efficiency
125	—/0	1.36	110.8
120	1.8/10	1.38	122.4
115	2/15	1.37	131.7
